# Docosahexaenoic Acid and Sleep Quality in Very and Extreme Preterm Infants

**DOI:** 10.3390/ijerph21101362

**Published:** 2024-10-15

**Authors:** Giovanna Rando Barion, Pietra Giovanna Marghetti, Patricia Zanotelli Cagliari, Marco Fabio Mastroeni

**Affiliations:** 1 Postgraduate Program in Health and Environment, University of Joinville Region, Rua Paulo Malschitzki, Joinville 89219-710, SC, Brazil; gibarion@gmail.com (G.R.B.); patriciacagliari@univille.br (P.Z.C.); 2 Nursing Department, University of Joinville Region, Rua Paulo Malschitzki, Joinville 89219-710, SC, Brazil; pietragiovanna3d@gmail.com; 3 Darcy Vargas Maternity Hospital, Joinville 89202-190, SC, Brazil; 4 Medicine Department, University of Joinville Region, Rua Paulo Malschitzki, nº 10, Joinville 89219-710, SC, Brazil

**Keywords:** children, sleep habits, preterm infant, docosahexaenoic acid, birth weight

## Abstract

The results regarding the association of plasma docosahexaenoic acid (DHA) levels with sleep duration conflict. This study aimed to investigate the effect of oral administration of DHA on the sleep quality of Brazilian extreme preterm infants. This cohort study is part of the Joinville Docosahexaenoic Acid Study (JoiDHA) conducted with 59 infants in Joinville, Brazil. Sleep quality was assessed using the Brief Infant Sleep Questionnaire, which consists of 12 questions about the quality of sleep the week prior to its application and was answered by the parents/guardians. Of the 59 children who participated in the study, 37 (62.7%) were supplemented with DHA and 22 (37.3%) did not receive DHA. The prevalence of poor sleep quality was higher among children with the weight status at birth <50th percentile (68.2%; *p* = 0.045) when compared to children ≥50th percentile. However, Poisson regression analysis showed that neither weight status at birth nor DHA use was associated with sleep quality, even after adjusting for the same variables. In summary, sleep quality 12–24 months after birth was not associated with DHA supplementation in very and extreme preterm infants. Additional studies that address the increase in DHA intake would be important for the understanding of the effect of this fatty acid on sleep quality.

## 1. Introduction

Prematurity is the main cause of death during the neonatal period and the second most common cause of pediatric death before the age of five [1]. In Brazil, the prevalence of preterm birth is approximately 10% [2], with a reduction in the average annual rate of 0.5% or more between 2010 and 2020 [3].

Preterm birth results in important short and long-term negative neonatal outcomes [4], including bronchopulmonary dysplasia [5], metabolic bone disease [6], retinopathy of prematurity [7], hearing difficulties [8], cognitive and behavioral changes [9], impairment of neurological development [9], and sleep disorders [10]. Sleep disorders can compromise physical health and the cognitive, motor, and psychological development of the individual throughout life [11]. Sleep, in turn, is essential for brain development. Preterm birth disrupts the normal ontogeny of sleep and leads to changes in its architecture, such as decreased total sleep time, reduced sleep efficiency, and sleep fragmentation [12]. The loss of the intrauterine environment and placenta, exposure to severe hypoxia or ischemia, and respiratory problems can negatively impact the sleep of preterm infants, significantly impairing their neurodevelopment [12].

In the first months of life, breastfeeding is important to minimize the negative effects of prematurity and to ensure the best development of the child, due to the presence of important components [13]. Among these components, the long-chain polyunsaturated fatty acids n-3 linolenic and n-6 linoleic are particularly important as precursors of docosahexaenoic acid (DHA) and arachidonic acid, which play an important role in the central nervous system [14]. DHA is also involved in the synthesis of melatonin, a hormone released by the pineal gland in the early evening that regulates the sleep cycle [15]. In fact, plasma DHA levels have been shown to be associated with sleep duration [16].

Few studies have addressed DHA and sleep. Some studies have investigated sleep patterns and sleep quality in preterm infants [17,18], but few have evaluated the effect of DHA on sleep quality in early childhood [19]. A randomized study of Japanese adults showed that daily supplementation with DHA and eicosapentaenoic acid for 12 weeks can improve sleep quality, with greater sleep efficiency compared to the placebo group [20]. Another study demonstrated that adequate maternal intake of fish rich in n-3 polyunsaturated fatty acids during pregnancy can reduce episodes of insufficient sleep in 12-month-old infants [21]. Since intrauterine accumulation of DHA occurs in the third trimester of pregnancy, preterm infants have low plasma concentrations and body reserves of this fatty acid [22]. Inadequate concentrations of DHA in preterm infants leads to a decrease in the body’s melatonin levels, compromising adequate neurodevelopment and sleep quality [14,16,21]. For example, an increase in awakenings is observed in the first days of life [14,21]. N-3 polyunsaturated fatty acids act on immune/inflammatory and neurological pathways, which are associated with circadian clock regulation [23]. The role of DHA in neurodevelopment is linked to its incorporation into neuronal membranes, affecting signal transduction and neurotransmitter function, which can influence sleep and circadian rhythms [24]. Thus, DHA supplementation is therapeutically beneficial for circadian disruption related pathologies [23] and might be a good alternative to prevent sleep disorders. Within this context, the aim of this study was to investigate the effectiveness of oral DHA supplementation in improving the sleep quality of Brazilian infants aged 12–24 months born very or extreme preterm. The results obtained in this study will contribute to a better understanding of the relationship between DHA and sleep quality. Our hypothesis is that DHA supplementation improves the sleep quality of Brazilian infants.

## 2. Materials and Methods

### 2.1. Study Design and Participants

This cohort study is part of the Joinville Docosahexaenoic Acid Study (JoiDHA Study, Brazil), a clinical trial study conducted at the Darcy Vargas Maternity (DVM) hospital, the only public maternity hospital in Joinville, Santa Catarina, Brazil, which is responsible for 60% of deliveries in the city. The JoiDHA Study proposed to evaluate the effect of DHA supplementation on retinopathy of prematurity and other child outcomes throughout life, including sleep quality.

**Figure 1:**
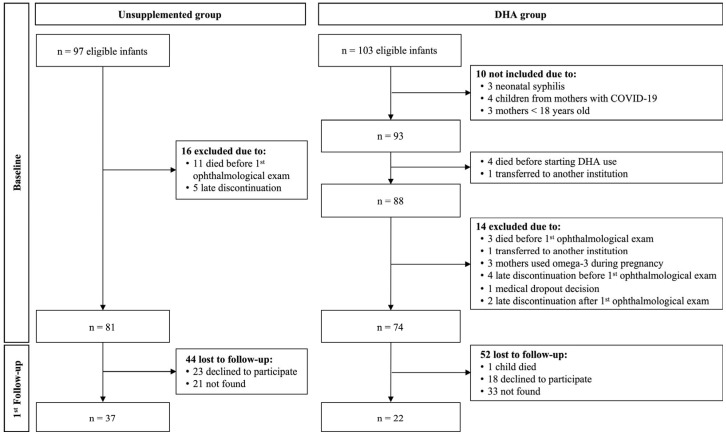
Flow diagram of the study. Adapted from Cagliari et al. [25].

Details of the JoiDHA Study recruitment process were published previously [25]. Briefly, the JoiDHA study included mothers older than 18 years and newborns up to 33 weeks of gestational age (up to 32 weeks + 6 days) and/or with a birth weight ≤1500 g seen at the DVM hospital. The participants were recruited at two different times and divided into two groups: unsupplemented group (1 March 2020 to 8 June 2021, *n* = 81), which did not receive DHA supplementation, and DHA group (9 June 2021 to 31 January 2023, *n* = 74), which received DHA supplementation (Figure 1). Infants with ocular malformations who died before the first ophthalmological examination, infants who were transferred to a hospital in another city, and infants whose mothers had been supplemented with DHA or had infectious diseases (toxoplasmosis, syphilis, or corona-virus disease 2019 [COVID-19]) were excluded [25]. Of the 81 and 74 eligible infants in the unsupplemented and DHA groups, respectively, 22 and 37 participated in this study (Figure 1).

The current study was conducted from 1 September 2022 to 20 August 2023 (DHA group) and all 155 individuals of the JoiDHA Study were invited to participate. This study also followed the Strengthening the Reporting of Observational Studies in Epidemiology (STROBE) statement.

### 2.2. JoiDHA Study: Sample Size, Data Collection, and DHA Supplementation

The sample size was performed using the G*Power software (version 3.1.9.6) by proportion test and for two independent groups. From a prevalence of retinopathy of prematurity of 40% [26,27], a confidence interval of 95%, an alpha error of 5%, a power of 80%, and losses of 10%, the expected number would be 180 individuals (unsupplemented and DHA groups) [25].

Data were obtained from the medical records at the maternity hospital (unsupplemented group) and by interview with the parents/guardian in a private room of the maternity hospital within 48 h after the child’s birth (DHA group). The data collected included sociodemographic, biological, clinical, and anthropometric measurements (mother’s age, education, marital status, monthly household income, type of delivery, sex, gestational age at birth, birth weight, length, and weight status at birth).

Birth length was measured to the nearest 0.1 cm with a pediatric anthropometric ruler (WCS^®^, Wood, Curitiba, PR, Brazil), and birth weight was measured to the nearest 1 g with a digital pediatric portable scale (Filizola^®^, BP Baby, São Paulo, SP, Brazil) using the same equipment throughout the study [25]. The birth weight was categorized as <1000 g and ≥1000 g according to the International Statistical Classification of Diseases and Related Health Problems (ICD-10) [28]. The weight status at birth was classified into two categories based on gestational age and sex, according to the INTERGROWTH-21st standards [29]: <50th and ≥50th percentile.

Details of the DHA supplementation were also published previously [25]. Concisely, DHA was extracted from fish oil capsules of a commercial supplement (Mega DHA^®^, VITAFOR, Sorocaba, SP, Brazil) composed of 57.0% DHA and 43.0% other fatty acids [25]. The oil of each capsule was transferred to 10 mL eyedrop bottles by a compounding pharmacy [25]. The bottles contained approximately 66.3 mg DHA/oil drop and were used at the maternity hospital during the study period and after hospital discharge, at home [25].

DHA supplementation began when the infant’s daily feed volume reached 100 mL/kg, between the 4th/6th day of life and was continued during the time the child remained in the maternity hospital. DHA was administered daily by the attending nurse directly into the mouth, immediately before feeding [25]. The infants received DHA supplementation according to its weight and regardless of the type of diet: <1000.0 g = 1 drop (66.3 mg DHA); 1000 to <2000.0 g = 2 drops (132.6 mg DHA); 2000 to <3000.0 g = 3 drops (198.9 mg DHA); 3000 to <4000.0 g = 4 drops (265.2 mg DHA); 4000 to <5000.0 g = 5 drops (331.5 mg DHA), and 5000 to <6000.0 g = 6 drops (397.8 mg DHA) [25].

After hospital discharge, the participants received one DHA eyedrop bottle free of charge and were instructed to continue DHA administration at home immediately before the regular diet until the next ophthalmological evaluation [25]. DHA supplementation was finished when complete vascularization of the peripheral retina was detected in the ophthalmological examination, accompanied every 15 days [25].

### 2.3. General Data Collection

Using the telephone and social media contacts obtained from the JoiDHA Study data, all mothers who participated in the JoiDHA Study were contacted individually and invited to participate in the study. Due to the risk of contamination with the COVID-19 virus, the data were collected through online interviews.

When the mothers/guardians answered or returned the contact, the researcher explained the objectives of the study and invited them to participate. Individuals who agreed to participate in the study were asked to schedule a date for an online interview according to their convenience. On the scheduled date, the mothers/guardians were contacted via social media using the video call feature and then received a copy of the informed consent form signed by the main researcher of the study. The participants were asked to sign the form and to send it back using an specific platform for digital signature. During the interview, the mothers/guardians were asked about the sociodemographic and biological data of the mother/child pair (mother’s age, education, marital status, and monthly household income). Guided by the researcher during the interview, anthropometric weight and height were measured by the mothers/guardians using their own scale and inextensible measuring tape.

Two measurements of each variable were carried out and the mean of these measurements was used as the final value.

### 2.4. Sleep Quality

Sleep quality was assessed using the Portuguese version of the Brief Infant Sleep Questionnaire (BISQ), which is designed to screen for sleep disorders in infants and children from 0–3 years of age [30]. The instrument was developed and validated in 2004, translated into Brazilian Portuguese in 2012 [30], and validated for Brazilian children in 2020 [31]. BISQ was chosen because of its good reliability, validity, and clinical applicability for the quick assessment of sleep problems in infants and toddlers for clinical and research purposes [31,32]. Furthermore, the instrument was also used in studies conducted in countries other than Brazil [33,34,35,36]. The high specificity (90.1–96.6%) of the BISQ sleep parameters supports the validity of parents’ statements on sleep-related problems in children up to 36 months of age for use in epidemiological studies [31].

The instrument consists of 12 questions referring to the week prior to its application, including the place where the child sleeps, the position in which the child sleeps, the total time spent sleeping at night and during the day, the time the child remains awake after falling asleep, sleep onset time, conditions for falling asleep, and whether sleep is considered a problem for parents [30]. The questionnaire was answered by the parents/guardians, which lasted approximately 5–10 min. Since the data come from a larger birth cohort study conducted from 1 March 2020 to 31 January 2023, the children were not born on the same day. Therefore, the children’s ages ranged from 12 to 24 months.

Sleep quality was classified into two categories where poor sleep quality was defined as when the child met at least one of the following criteria: (1) waking up 4 times/night or more; (2) nighttime awakenings longer than 1 hour, and (3) total sleep time less than 9 h [32]. Adequate sleep was defined as when the child did not meet any of the three criteria.

The weight status was assessed using the body mass index (BMI), calculated by dividing weight by the square of height [weight (kg)/height (m^2^)], and was classified into two categories according to the WHO Child Growth Standards [37]: ≤85th and >85th percentile.

### 2.5. Statistical Analysis

The IBM SPSS Statistics for Macintosh program, version 29.0 (Released 2022, IBM Corp., Armonk, NY, USA), was used to analyze the data. Categorical variables were compared using the χ^2^ test. The sleep characteristics according to DHA use are expressed as median and interquartile range. Continuous variables were compared between the JoiDHA study participants at birth and the current study using the Mann–Whitney *U* test (online Supplementary Material Table S1).

Poisson regression analysis was performed to examine potential associations between the predictors and the outcome (sleep quality). The relative risk (RR) and 95% confidence interval (CI) were calculated. The variables included in the unadjusted model were selected based on the association between each predictor and the outcome (Model 1). Variables with *p* < 0.1 by the χ^2^ test were included in the adjusted models (weight status at birth and DHA use). DHA use was included in the adjusted analysis, since this variable was the main predictor of the study and because of the interest in evaluating its effect. The “Enter” method including each variable in the model was chosen to construct the adjusted model (Model 2).

The −2log-likelihood value was used to assess the goodness-of-fit of the models, with the lowest value indicating the best model. The results were considered statistically significant when *p* < 0.05.

## 3. Results

### 3.1. Characteristics of Participants

Supplementary Material Table S1 shows the comparison of biological variables between participants at birth and those lost to follow-up in the current study. There was no significant (*p* > 0.05) difference in mother’s age at delivery, gestational age at birth, birth weight, or birth length between the two groups (online Supplementary Material Table S1). Descriptive characteristics of the child’s sleep quality according to BISQ are shown in online Supplementary Material Table S2.

Table 1 shows the sociodemographic and biological characteristics of the participants according to sleep quality. About 31.8% of children with weight status at birth <50th percentile had poor sleep quality (*p* = 0.045). However, although the difference between the groups was significant, the effect size was small (Phi = 0.261), indicating a small magnitude of the true difference between the sleep quality groups considering weight status at birth.

### 3.2. Sleep Quality Characteristics

The sleep characteristics according to DHA use and the child’s sleep quality are described in Table 2. Although the median sleep time’s minutes were higher for infants in the DHA group (except for sleep time at night), this difference was not significant (*p* > 0.05) when compared to the unsupplemented group (Table 2).

### 3.3. Determinants of Sleep Quality

The models explaining the determinants of sleep quality in infants are presented in Table 3. Poisson regression analysis showed that neither weight status at birth nor DHA use was associated (*p* > 0.05) with sleep quality (Model 1, Table 3), even after adjusting for the same variables (Model 2, Table 3).

## 4. Discussion

The present study showed that sleep quality 12–24 months after birth is not associated with DHA use in very and extreme preterm Brazilian infants.

The results of studies evaluating the relationship between sleep quality and DHA supplementation are still controversial [16,19,20]. In agreement with our results, some authors found no relationship between sleep quality and DHA supplementation [19], while others demonstrated an association between plasma DHA levels and sleep duration [16] and between DHA supplementation and sleep duration [19,21].

Preterm children are vulnerable to different conditions because of the incomplete development of physiological, metabolic, and anatomical characteristics [9]. Since very preterm infants are born before or in the last trimester of pregnancy, DHA is not transferred from the mother in adequate quantities to meet the infants’ needs [14], who therefore have low levels of circulating DHA [16]. These low DHA levels affect melatonin synthesis, which is associated with sleep quality [16,38,39]. On the other hand, the transfer of an appropriate concentration of DHA to the baby in the last trimester of pregnancy leads to an increase in brain DHA levels and melatonin levels in the body, contributing to adequate neurodevelopment and sleep quality [14,16,21]. Within this context, increasing melatonin concentrations in the body promotes earlier sleep onset and longer total sleep time [16,38,39], benefiting the newborn’s sleep by reducing awakenings in the first days of life [14,21]. Thus, supplementing very and extreme preterm infants with DHA after birth may be a promising alternative to minimize its deficiency, especially in the first weeks of life.

We understand that several factors can influence the association between DHA and sleep quality, including DHA dose, sample size, and study design, among other factors. Unlike other studies [20,40,41], DHA supplementation was extended beyond hospital discharge in our study, with administration at home. Nevertheless, despite longer DHA supplementation, our results showed no effect on sleep quality. We also used supplementation with oral DHA drops immediately before breastfeeding because of the better absorption of DHA considering the infant’s immature gastrointestinal tract. The adoption of breastfeeding immediately after DHA supplementation may also stimulate the growth of Bifidobacteriae and *Lactobacillus* species, thus contributing to gastrointestinal absorption [42]. The differences in DHA dose also impair the comparison between studies. In general, a DHA dose of 40 to 120 mg/kg/day was administered to the infant [40,43,44,45], similar to the concentration of 66.3 mg/kg/day used in our study.

It is also important to comment on the effect of weight status at birth on the child’s sleep quality. The relationship between low birth weight and sleep quality is still controversial in the scientific literature. Although our results of adjusted Poisson regression analysis indicated a borderline association (*p* = 0.052) between weight status at birth and sleep quality, some authors found an association of low birth weight and preterm birth with sleep disturbances and poor sleep quality [46,47]. In contrast, another study showed that growth restriction in fetal and infant stages promotes longer sleep duration and higher sleep efficiency at school age [48]. This finding might be due to increased sleep requirements for maturational processes and development after a difficult very preterm birth [48].

In addition to the controversial results regarding weight status at birth and sleep quality reported in the scientific literature, we believe that the COVID-19 pandemic had a potential confounding effect on the study since it may have affected the quality of sleep of both the mother before and/or after childbirth and the child. Many psychological problems including poor sleep health may have emerged during the COVID-19 pandemic [49]. A study comparing infant and toddler sleep patterns before and during the first wave of home restriction due to COVID-19 in Spain revealed a later bedtime, as well as a significant increase in infant and toddler sleep latency of >30 min during confinement [50].

In summary, more studies investigating DHA-rich foods and DHA supplementation, as well as serum DHA levels, are essential to understand its effect on sleep quality, especially in children. New studies involving newborns should consider the dosage of DHA and also other complex and intrinsically associated variables such as how DHA is supplemented, whether before breastfeeding or immediately thereafter, whether the child received artificial or human milk, whether supplementation was given orally directly into the mouth or enterally, oxygen supply, gestational age, and the mother’s weight status, among other characteristics associated with the absorption of DHA by the still-developing gastrointestinal system of the child.

This study has some strengths. First, the study used primary data collected by the team that conducted the study. Second, the type of oral supplementation used in our study is an interesting low-cost method that can be easily applied to infants and can potentially be used in other situations to overcome DHA deficiency in newborns and infants.

The present results can be applied to clinical studies that address other outcomes. Although this study did not find a significant effect of DHA use on sleep quality in children aged 12–24 months, oral DHA supplementation can be used to assess various clinical outcomes even after hospital discharge. This is an important characteristic to ensure the success of treatments outside the hospital environment in the patient’s own home. In addition, sleep quality is only one outcome that can potentially be affected by DHA supplementation at birth. Other outcomes such as retinopathy of prematurity and cognitive development also deserve attention and further investigation.

Some limitations of the study should also be mentioned. First, the relatively small sample size limited the multivariate analysis and the investigation of other important covariates. Second, the interviews were carried out online and the anthropometric data were collected by the mothers, a fact that may have influenced these measurements. Third, the small number of participants may also explain the lack of correlation between DHA and sleep quality. Therefore, studies with a larger number of individuals will probably be able to answer whether there is a relationship between DHA and sleep quality. Lastly, the COVID-19 pandemic made it difficult to approach mothers in person, compromising data collection.

## 5. Conclusions

In conclusion, we found that sleep quality 12–24 months after birth is not associated with DHA supplementation in very preterm and extreme preterm infants. Further studies that address the increase in DHA levels as a result of supplementation or DHA-rich foods in preterm infants would be important for understanding the effect of DHA on sleep quality and neurodevelopmental problems.

## Figures and Tables

**Table 1:** Sociodemographic and biological characteristics of the study participants according to the child’s sleep quality. JoiDHA Study, Brazil, 2020–2023 (*n* = 59).

Characteristic	Sleep Quality			
	Adequate (*n* = 48)	Poor (*n* = 11)	Total (*n* = 59)	*p*-Value ^a^
	*n* (%)	*n* (%)	*n* (%)	
Mothers				
Age (years)				0.476
<30	17 (89.5)	2 (10.5)	19 (32.2)	
≥30	31 (77.5)	9 (22.5)	40 (67.8)	
Education (years of schooling)				0.953
≥12	31 (81.6)	7 (18.4)	38 (64.4)	
<12	17 (81.0)	4 (19.0)	21 (35.6)	
Marital status				0.670
Married/consensual union	38 (79.2)	10 (20.8)	48 (81.4)	
Other	10 (90.9)	1 (9.1)	11 (18.6)	
Monthly household income (MW)				0.883
≥3	23 (82.1)	5 (17.9)	28 (47.4)	
<3	25 (80.6)	6 (19.4)	31 (52.5)	
Children				
Sex				0.325
Male	26 (76.5)	8 (23.5)	34 (57.6)	
Female	22 (88.0)	3 (12.0)	25 (42.4)	
Type of delivery				0.721
Normal	19 (79.2)	5 (20.8)	24 (40.7)	
Cesarean	29 (82.9)	6 (17.1)	5 (59.3)	
Gestational age at birth (weeks)				0.944
≥28	30 (81.1)	7 (18.9)	37 (62.7)	
<28	18 (81.8)	4 (18.2)	22 (37.3)	
Birth weight (g)				0.900
≥1000	24 (81.0)	8 (19.0)	42 (71.2)	
<1000	14 (82.4)	3 (17.6)	17 (28.8)	
Weight status at birth (percentile) ^b^				0.045
≥50th	33 (89.2)	4 (10.8)	37 (62.7)	
<50th	15 (68.2)	7 (31.8)	22 (37.3)	
Body mass index (percentile) ^c^				0.269
≤85th	35 (77.8)	10 (22.2)	45 (76.3)	
>85th	13 (92.9)	1 (7.1)	14 (23.7)	
DHA use				0.535
Yes	17 (77.3)	5 (22.7)	22 (37.3)	
No	31 (83.8)	6 (16.2)	37 (62.7)	

MW: minimum wage (US $269.00 in 2023); DHA: docosahexaenoic acid. ^a^ *p*-Value refers to the *X*^2^ test (statistical significance at *p* < 0.05). ^b^ INTERGROWTH-21st standards. ^c^ 2006 WHO growth standards.

**Table 2:** Sleep characteristics according to DHA use and the child’s sleep quality (Joinville, Brazil, 2022–2023).

Characteristic	Adequate Sleep Quality			*p*-Value ^a^	Poor Sleep Quality			*p*-Value ^a^
	Unsupplemented (*n* = 31)	DHA Use (*n* = 17)	Total (*n* = 48)		Unsupplemented (*n* = 6)	DHA Use (*n* = 5)	Total (*n* = 11)	
	Median (IQR)	Median (IQR)	Median (IQR)		Median (IQR)	Median (IQR)	Median (IQR)	
Time spent sleeping at night (min)	600.0 (105.0)	540.0 (150.0)	600.0 (105.0)	0.790	390.0 (260.0)	540.0 (150.0)	420.0 (180.0)	0.164
Time spent sleeping during the day (min)	90.0 (60.0)	120.0 (120.0)	105.0 (60.0)	0.326	50.0 (75.0)	90.0 (120.0)	60.0 (90.0)	0.269
Total sleep time (min)	660.0 (120.0)	720.0 (190.0)	660.0 (165.0)	0.836	470.0 (185.0)	570.0 (240.0)	510.0 (170.0)	0.067
Time awake after falling asleep (min)	0	0	0	0.462	0	20.0 (35.0)	0 (20.0)	0.090
Sleep onset time (min)	10.0 (25.0)	10.0 (10.0)	10.0 (15.0)	0.243	30.0 (18.0)	10.0 (58.0)	30.0 (30.0)	0.305

DHA: docosahexaenoic acid; IQR: interquartile range. ^a^ *p*-Value refers to the Mann–Whitney *U* test (statistical significance at *p* < 0.05).

**Table 3:** Predictors of the child’s sleep quality. JoiDHA Study, Brazil, 2020–2023 (*n* = 59).

Characteristic	Model 1		Model 2	
	RR (95% CI)	*p*-Value	RR (95% CI)	*p*-Value
Weight status at birth (percentile) ^a^				
≥50th	Reference		Reference	
<50th	2.94 (0.97; 8.92)	0.056	2.96 (0.98; 8.93)	0.054
DHA use				
Yes	Reference		Reference	
No	0.71 (0.24; 2.07)	0.534	0.70 (2.52; 1.95)	0.498

RR: relative risk; 95% CI: 95% confidence interval; DHA: docosahexaenoic acid. *p*-value refers to the Wald test in Poisson regression. Model 1: unadjusted relative risk. Model 2: relative risk adjusted for weight status at birth and DHA use. ^a^ INTERGROWTH-21st standards.

## Data Availability

Data will be made available upon request to the authors and through a formal data sharing agreement.

## Supplementary Materials

The following supporting information can be downloaded at: https://www.mdpi.com/article/10.3390/ijerph21101362/s1, Table S1: Losses to follow-up from birth to the current study (JoiDHA Study, Brazil, 2020–2023); Table S2: Descriptive characteristics of the BISQ instrument according to the child’s sleep quality. JoiDHA Study, Brazil, 2020–2023.
