# Supplementary material for: Docosahexaenoic Acid and Sleep Quality in Very and Extreme Preterm Infants
Source: Int J Environ Res Public Health. 2024 Oct 15;21(10):1362. doi: 10.3390/ijerph21101362 (PMC11507004; doi:10.3390/ijerph21101362)
Supplement: Supplementary file 1 [file ijerph-21-01362-s001.zip › ijerph-3179635-supplementary.pdf]

Table S1. Losses to follow-up from birth to the current study (JoiDHA Study, Brazil, 2020-2023).

Characteristic	Study participants		*p-Value**
	Losses to follow-up (n = 96)	Current study (n = 59)	
	Median (IQR)	Median (IQR)	
Mother's age at delivery (years)	29.0 (12.0)	31.0 (8.0)	0.385
Gestational age at birth (weeks)	30.0 (3.0)	30.0 (4.0)	0.674
Birth weight (g)	1,275.0 (675.0)	1,258.0 (620.0)	0.325
Length (cm)	38.0 (5.0)	38.0 (6.0)	0.405

IQR, interquartile range. * Mann-Whitney *U* test.

Table S2. Descriptive characteristics of the BISQ instrument according to the child's sleep quality. JoiDHA Study, Brazil, 2020-2023.

Characteristic	Sleep quality (n = 59)	
	Adequate (n = 48)	Poor (n = 11)
	n (%)	n (%)
Birth order of the child		
Oldest	19 (39.6)	6 (54.5)
Middle	4 (8.3)	1 (9.1)
Youngest	25 (52.1)	29 (36.4)
Sleeping arrangement		
Infant crib in a separate room	10 (20.8)	3 (27.3)
Infant crib in parents' room	38 (79.2)	8 (72.7)
Sleeping position		
On his/her belly	11 (22.9)	4 (36.4)
Other	37 (77.1)	7 (63.6)
Number of night wakings per night (n)		
0	25 (52.1)	6 (54.5)
≥ 1	23 (47.9)	5 (45.5)
Do you consider your child's sleep a problem?		
No	38 (79.2)	5 (45.5)
Yes	10 (20.8)	6 (54.5)
	Mean (SD)	Mean (SD)
Time spent sleeping at night (h)	9.5 (0.2)	7.2 (0.8)
Time spent sleeping during the day (h)	1.7 (0.1)	1.3 (0.3)
Total sleep time (h)	11.3 (0.2)	8.5 (0.9)
Time awake after falling asleep (min)	10.1 (2.2)	16.9 (2.2)
Sleep onset time (min)	13.7 (1.3)	30.4 (9.9)

SD, Standard deviation.
